# Modulation of Antibacterial, Antioxidant, and Anti-Inflammatory Properties by Drying of *Prunus domestica* L. Plum Juice Extracts

**DOI:** 10.3390/microorganisms8010119

**Published:** 2020-01-15

**Authors:** Jose Manuel Silvan, Anna Michalska-Ciechanowska, Adolfo J. Martinez-Rodriguez

**Affiliations:** 1 Microbiology and Food Biocatalysis Group, Department of Biotechnology and Food Microbiology, Institute of Food Science Research (CIAL), CSIC-UAM-C/Nicolás Cabrera, 9. Cantoblanco Campus, Autonoma University of Madrid, 28049 Madrid, Spain; 2 Department of Fruit, Vegetable and Plant Nutraceutical Technology, the Faculty of Biotechnology and Food Science, Wrocław University of Environmental and Life Sciences, Chełmońskiego 37, 51-630 Wrocław, Poland; anna.michalska@upwr.edu.pl

**Keywords:** *Prunus domestica* L., plum extracts, drying, polyphenolics, bioactive properties, antibacterial, antioxidant anti-inflammatory

## Abstract

The consumption of plums in a fresh form is seasonal, therefore the transformation of plum juice extracts into powdered form is a good alternative for its longer availability throughout the year. The drying process can moderate the physical and chemical properties of the plum extracts, thus, this study examined the changes in biological activity, i.e., antibacterial, antioxidant, and anti-inflammatory properties moderated by freeze, vacuum, and spray drying. It was suggested that the drying processes and the applied parameters might moderate the content of polyphenolic compounds in the powders, which influence the different levels of growth inhibition against the foodborne pathogens (17% to 58% of inhibition), demonstrating a strain-dependent effect. These powders could also induce cellular protection against oxidative stress by preventing intracellular reactive oxygen species (ROS) accumulation (23% to 37% of reduction), but the level of antioxidant capacity may be determined by the conditions applied during the drying process. Moreover, plum extract powders exhibited a greater anti-inflammatory capacity (24% to 39% of inhibition), which would be influenced both, by the type of treatment used and by the temperature used in each treatment. The results demonstrate that the selection of the drying method can be an effective tool for modulating the composition, physical, and bioactive properties of plum extracts powders.

## 1. Introduction

Diets rich in fruits are beneficial to human health because of their polyphenolic compound content. In this regard, plums (*Prunus domestica* L.) represent an excellent source of such components, which can contribute significantly to the prevention of several diseases [1]. This fruit is cultivated all over the world and its production in the last 10 years has exceeded 11 million tons [2]. Plum is a seasonal fruit, and the harvest period and the period of supply of fresh fruit are relatively short. Therefore, plums cannot be consumed fresh throughout the year, thus, the development of new dried powder products, obtained by drying industrial techniques, offers an alternative for the consumption at any season of the year. Plum powders can be obtained from whole fruit [3], plum by-products [4], or juices/concentrates [5]. Commonly used drying industrial processes include, apart from conventional air-drying, freeze-drying (FD), vacuum drying (VD), and spray drying (SPD) [6]. However, the drying techniques, used to obtain powders from whole fruit, modify some of their physical and chemical properties [3,7]. In general, FD is considered one of the best method of obtaining high-quality products because the absence of liquid water and the low temperatures required for the process allows relatively high retention of bioactive compounds [8]. VD partially prevents thermal degradation of bioactive compounds in raw material because the temperature of the product is usually low and can be easily controlled [9]. And SPD is an effective technique in drying liquid products directly into powders, and is used broadly in processing dairy and fruit products, i.e., juices, extracts, or concentrates [10], and the resulting powders present a better preservation and retention of polyphenolic compounds [11,12]. Numerous studies confirmed that the above-mentioned processes had a strong influence on the physical properties of the dried whole fruits and pomace [3,5,13], as well as of the fruit juice powders [12].

Plums are phenolic-rich fruits that contain a mixture of polyphenolic compounds that can exert several biological effects, including antibacterial [14,15], antioxidant [5,16], and anti-inflammatory properties [17,18]. In commercially available plums, the most predominant and bioactive relevant compounds are phenolic acids, such as chlorogenic and neochlorogenic acids; flavonol glycosides, quercetin-3-glucoside and quercetin-3-galactoside; and anthocyanins, such as cyanidin and peonidin [19]. However, the profile and level of bioactive polyphenolic compounds in dried plum products is affected by the drying industrial processes. Until now, there is a lack of information in the literature on how the drying processes can modify the biological properties of fruit powders, regardless of the type of the fruit used for drying. Therefore, the objective of the present study was to evaluate the influence of different drying techniques applied for preparation of plum juice extract powders on the physical properties and the alterations in polyphenolic compounds contents and bioactive properties, i.e., antibacterial, antioxidant, and anti-inflammatory.

## 2. Materials and Methods

### 2.1. Reagents

3,4,5-Dimethylthiazol-2,5-diphenyl-tetrazolium bromide (MTT), carboxy-2′,7′-dichloro-dihydro-fluorescein diacetate (DCFH-DA), dimethyl sulfoxide (DMSO) were acquired from Sigma (Madrid, Spain). Dulbecco’s Modified Eagle’s Medium (DMEM), penicillin/streptomycin (5000 U/mL), phosphate buffered saline (PBS) and trypsin/EDTA solution (170,000 U/L) were purchased from Lonza (Madrid, Spain). Fetal bovine serum (FBS) of South American origin (Hyclone, GE Healthcare, Logan, UK) was obtained from Thermo Scientific (Madrid, Spain). Cell culture dishes were obtained from Sarstedt (Barcelona, Spain).

### 2.2. Materials

Material used in the study consisted of plum (*Prunus domestica* L.) (cv. Valor) juice obtained by laboratory hydraulic press (SRSE, Warsaw, Poland) that was used for preparation of polyphenolic extract. This was done by application of amberlite XAD-16 resin previously washed with water. The absorbed compounds were removed by ethanol that was evaporated by rotary evaporator Laborota 20 (Heidolph, Schwabag, Germany) at 40 °C to avoid the excessive degradation of the polyphenols. The solution was divided into 100 mL portions and subjected to the drying processes without the addition of any carrier agent due to the sugars’ removal [5].

### 2.3. Drying Procedure

The plum juice extracts were submitted to drying techniques: (a) freeze-drying (FD) was performed in freeze-dryer (FreeZone, Labconco Corp., Kansas City, MO, USA) for 24 h (temperature of the chamber −60 °C and the heating plate +25 °C); (b) vacuum drying (VD) in Vacucell 111 Eco Line (MMM Medcenter Einrichtungen GmbH, Planegg, Germany) at the temperature of 40 °C, 60 °C and 80 °C at a pressure of 300 Pa for, respectively, 20, 16, and 10 h; (c) spray drying (SPD) done by mini spray dryer (Buchi, Flawil, Switzerland) with the inlet temperature of 180 °C and the outlet temperature of 70 °C. All drying techniques were performed at least in duplicate (*n* = 2). The obtained plum extracts powders (PEP) were milled (Bosch MKM 6003c, Gerlingen, Germany), vacuum packed (PP-5.14, Tepro SA, Koszalin, Poland), and stored at −20 °C until they were analyzed.

### 2.4. Physicochemical Properties

The moisture content of PEP was determined in duplicate (*n* = 2) according to Figiel et al. [20] using Vacucell 111 Eco Line at 80 °C for 20 h. The results were expressed as %. The water activity of the PEP was done at 25 °C by Water Activity Meter AquaLab (DewPoint 4Te, Decagon Devices Inc., Pullman, WA, USA) (*n* = 2). The colour of the samples was determined in a CIE L*a*b* system using a Minolta Chroma Meter CR-400 (Minolta Co. Ltd., Osaka, Japan). Data were presented as an average value of five measurements (*n* = 5).

### 2.5. Characterization of Polyphenolic Compounds by UPLC-PDA System

The preparation of the PEP extracts subjected to characterization of polyphenolic compounds using the UPLC-PDA system was performed according to Wojdyło et al. [21]. All determinations were done in duplicate. The results were expressed as grams per 100 g of dry basis (db) of plum extract power (g/100 g db).

### 2.6. Bacterial Strains, Growth Media, and Culture Conditions

Five of the most relevant foodborne pathogen bacteria were tested for antibacterial activity of PEP: *Campylobacter jejuni* NCTC11168, *Escherichia coli* ATCC^®^25922™, *Staphylococcus aureus* ATCC^®^25923™, *Listeria monocytogenes* CECT935, and *Salmonella enterica* subsp. enterica serovar Typhimurium ATCC^®^ 14028™. All bacteria strains were stored at −80 °C in Brucella Broth (BB) (Becton, Dickinson, & Co, Madrid, Spain) plus 20% glycerol. The agar-plating medium consisted of Müeller-Hinton agar supplemented with 5% defibrinated sheep blood (MHB) (Becton, Dickinson, & Co), and liquid growth medium consisted of BB. Bacteria cultures were prepared as follows: The frozen stored strains were reactivated by inoculation in MHB and incubation under aerobic conditions (*E. coli*, *S. aureus*, *L. monocytogenes*, and *S. typhimurium*) and microaerophilic conditions (*C. jejuni*) using a Variable Atmosphere Incubator (85% N_2_, 10% CO_2_, 5% O_2_) (VAIN, MACS-VA500, Don Whitley Scientific, Bingley, UK) at 37 °C for 24–48 h. Isolated colonies were inoculated into 50 mL of BB and incubated in the conditions described above following stirring (150 rpm) for 24–48 h until the late exponential phase, and used as experimental inoculum. These bacterial inoculum cultures ~1 × 108 colony forming units (CFU/mL) were used for the different experimental assays.

### 2.7. Antibacterial Activity

The antibacterial activity of PEP against foodborne bacteria was evaluated following the procedure described by Silvan et al. [22]. Briefly, 1 mL of PEP (1 mg/mL final concentrations) was transferred into different flasks containing 4 mL of BB. Bacterial inoculum (50 µL of ~1 × 10^8^ CFU/mL) was then inoculated into the flasks under aseptic conditions. The culture was prepared in triplicate and incubated under stirring (150 rpm) during 24 h at 37 °C. Growth controls were prepared by transferring 1 mL of sterile water to 4 mL of BB and 50 µL of bacterial inoculum. After incubation, serial decimal dilutions of mixtures were prepared in saline solution (0.9% NaCl) and they were plated (20 µL) onto fresh MHB agar and incubated as previously described. The number of CFU was assessed after incubation. The results of antibacterial activity were expressed as percentage of growth inhibition for the foodborne bacteria respect to the controls of bacteria growth.

### 2.8. Cell Cultures

Human intestinal epithelial HT-29 and murine macrophage RAW 264.7 cell lines were obtained from the American Type Culture Collection (ATCC). Both cell lines were cultured in Dulbecco’s modified Eagles’s medium (Lonza) supplemented with 10% fetal bovine serum (Hyclone) and 1% penicillin/streptomycin (5000 U/mL, Lonza). The cells were plated at densities of ~1 × 10^6^ cells in 75 cm^2^ culture flasks and maintained at 37 °C under 5% CO_2_ in a humidified incubator until 90% confluence. The culture medium was changed every 2 days. Before a confluent monolayer appeared, sub-culturing cell process was carried out.

### 2.9. Cell Viability

Before the cellular antioxidant and anti-inflammatory experiments, it was necessary to find out if the plum powders were cytotoxic for both cell lines (HT-29 and RAW 264.7). With this purpose, cell viability was determined by MTT reduction assay as previously was described by Silvan et al. [23]. Confluent stock cultures (~90%) were trypsinized (Trypsin/EDTA), and cells were seeded in 96-well plates (~5 × 10^4^ cells per well) and incubated in culture medium at 37 °C under 5% CO_2_ in a humidifier incubator for 24 h. Briefly, cell medium was replaced with serum-free medium containing PEP (1 mg/mL final concentrations), and the cells were incubated at 37 °C for 24 h under 5% CO_2_. Control cells (non-treated) were incubated in serum-free medium without plum powders. Thereafter, cells were washed with PBS, the medium was replaced by 200 μL of serum-free medium, and 20 μL of MTT solution in PBS (5 mg/mL) was added to each well for the quantification of the living metabolically active cells after 1-h incubation. MTT is reduced to purple formazan in the mitochondria of living cells. Formazan crystals in the wells were solubilized in 200 μL DMSO. Finally, the absorbance was measured at 570 nm wavelength by employing a microplate reader Synergy HT (BioTek Instruments Inc., Winooski, VT, USA). The viability was calculated considering controls containing serum-free medium as 100% viable. Data represent the mean and standard deviation of three independent experiments (*n* = 3). All experiments were carried out between passage 10 to passage 30 to ensure cell uniformity and reproducibility.

### 2.10. Antioxidant Activity Against Intracellular Reactive Oxygen Species (ROS) Production

Human intestinal epithelial cell line HT-29 was used for the evaluation of oxidative stress. Intracellular ROS were measured by the DCFH-DA assay as previously was reported by Martín et al. [24]. Cells were seeded (5 × 10^4^ cells per well) in 24-well plates and grown until they reached 70% of confluence. Cells were pre-treated with plum powders (1 mg/mL) dissolved in serum-free medium for 24 h. After that, the cells were washed with PBS and incubated with 20 μM DCFH-DA for 30 min at 37 °C. Then, cells were washed twice with PBS to remove the unabsorbed probe and treated with serum-free medium, containing 2.5 mM tert-butyl hydroperoxide (TBHP). ROS production was immediately monitored for 180 min in a fluorescent microplate reader Synergy HT using a λ_ex_ 485 nm and λ_em_ 530 nm. After being oxidized by intracellular oxidants, DCFH-DA changes to dichlorofluorescein (DCF) and emits fluorescence. The cells treated with TBHP was used as oxidative control (100% of intracellular ROS production). All samples were analyzed in triplicate (*n* = 3). All experiments were carried out between passage 10 to passage 30 to ensure cell uniformity and reproducibility. Data were expressed as percentage of fluorescence generation relative to negative control cells.

### 2.11. Anti-Inflammatory Activity

For inflammatory experiments, murine macrophage cell line RAW 264.7 was used. Cells were seeded in a 96-well plate at density of ~5 × 104 cells/well. Nitrite accumulation, indicator of nitric oxide (NO) synthesis, was measured in the culture medium of treated and control cells by the Griess reaction. After 24 h of incubation, the medium was removed, the cells were washed with 200 µL PBS and treated with PEP (1 mg/mL) and 10 µg/mL of LPS from *Escherichia coli* O55:B5 for 24 h. Control cells were incubated in serum-free medium with LPS (Control+) and without LPS (Control−) for 24 h. Finally, the media were collected and used for NO quantification. Briefly, 100 μL of collected cell supernatants were plated in 96-well plate and an equal amount of Griess reagent constituted by 1% (*w*/*v*) sulfanilamide and 0.1% (*w*/*v*) N-1-(naphthyl) ethylenediamine-diHCl in 2.5% (*v*/*v*) H_3_PO_4_, was added. The plate was incubated for 5 min and the absorbance measured at 550 nm in a microplate reader Synergy HT. The amount of NO was calculated using a sodium nitrite standard curve (0–10 µg/mL). Data were expressed as percentage of NO production calculated relative to Control+. Data represent the mean and standard deviation of three independent experiments (*n* = 3). All experiments were carried out between passage 10 to passage 30 to ensure cell uniformity and reproducibility.

### 2.12. Statistical Analysis

The results were reported as means ± standard deviations (SD) performed in triplicate. A *t*-test was used to assess the differences in antibacterial activity. Significant differences among the data were estimated by applying analysis of variance (ANOVA). The Tukey’s least significant differences (LSD) test was used to evaluate the significance of the analysis. Differences were considered significant at *p* < 0.05. All statistical tests were performed with IBM SPSS Statistics for Windows, Version 25.0 (IBM Corp., Armonk, NY, USA).

## 3. Results and Discussion

### 3.1. Physical Properties

The powders obtained from plum juice extracts submitted to different drying techniques differed in terms of moisture content and water activity (Table 1). The moisture content ranged from 2.44% up to 7.34% and was within the range described by Boonyai et al. [25]. The values of moisture content depended on the drying method and the parameters applied. The lowest water content was noted after spray drying during which the highest temperature for water removal was applied [12]. The evaluation of water activity in powders is a key aspect as this parameter informs about their stability, both chemical and microbial. It is connected with the quality of the dried products as the rate for some chemical reactions begins above a water activity of 0.3 [26]. Similarly, a high positive correlation (*r* = 0.789) between moisture content and water activity have been previously described in fruit powders [12]. PEP also differed in color attributes (Table 1).

Among products obtained, lower values of coordinate L* were noted after VD, when compared to FD and SPD, that was in agreement with previously conducted research on chokeberry [11] and apple powders [12]. The L* values were connected with coordinate a* and b* pointing a strong influence of the drying technique on the retention of red and yellow pigments. Chroma (C*) is connected with the color intensity and opposite to apple powders [12], the highest values were linked to FD and SPD processes. This could be connected with the fact that, in the current study, the extract of plum juice was dried, and thereby the material differed in terms of the chemical composition, as the obtained extracts were significantly darker when compared to the juice. Taking the above into consideration, the chemical changes that have occurred during drying are strictly connected with the chemical properties of the initial materials. The hue angle (h*) values indicated that the analyzed samples were more reddish as an angle of approximately 0 represents red colour. The highest h* values were obtained for powders gained after FD that was in line with the coordinate a*. Additionally, the lowest values were noted after FD and VD 40 °C, pointing to a strong influence on the temperature of the process on this parameter.

### 3.2. Polyphenolic Compounds Composition

The total polyphenolic compounds content in PEP ranged from 34.66 to 47.65 g/100 g db and differed due to the drying technique and parameters applied for their dehydration (Table 2) [5]. In general, the highest total content of identified polyphenolic compounds was noted after SPD, which was almost 19% higher when compared to FD, and is regarded as the technique that preserve bioactive compounds the most. Similarly, the highest retention of polyphenolic compounds in sugar-free extracts, submitted to SPD, was noted in case of chokeberry [11] and cranberry [27], which pointed to this dehydration method as being successfully used when the retention of selected polyphenolic compounds is concerned.

Going into the details, the major group of polyphenolic compounds present in PEP consisted of five identified phenolic acids, among which the chlorogenic and 3-feruoylquinic acids were dominant. The highest retention of these constituents was noted after SPD. As previously observed [5], the drying processes led to the degradation of phenolic acids within the increase of the temperature during VD, except methyl 3-caffeoylquinate. Interestingly, the increase in the temperature during VD up to 80 °C, followed by SPD (170 °C) resulted in, respectively, 29-, and 16-times higher content of this compound in the powders when compared to those gained after FD. In case of flavonols, processing during which the temperature above 40 °C (VD 40 °C, VD 60 °C, VD 80 °C and SPD) caused a strong degradation of quercetin-3-*O*-galactoside, the application of such conditions caused an increase in the content of quercetin-3-*O*-glucoside when compared to FD. This might be connected with the thermally-induced release of this constituent during drying. In general, the SPD process resulted in better retention of flavonols being almost 20% higher when compared to FD. In the current study, three anthocyanins had been identified and their content followed the same patterns as the above-mentioned groups of polyphenolic compounds.

### 3.3. Antibacterial Activity

**Figure 1:**
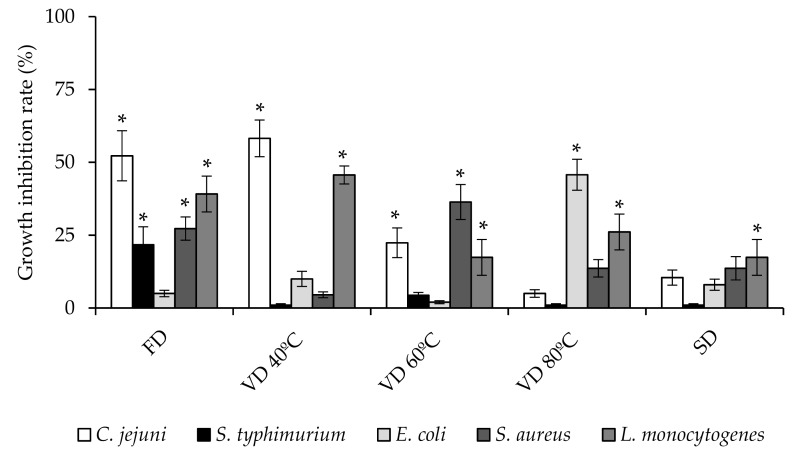
Effect of PEP (1 mg/mL) on foodborne bacteria growth after 24 h of incubation. Results represent the percentage of growth inhibition respect to the untreated control (100% of growth) and are expressed as mean ±SD (*n* = 3). Bars marked with asterisk indicate significant growth inhibition respect to the control by *t*-test (*p* ≤ 0.05). Freeze drying (FD); Vacuum drying 40 °C (VD 40 °C), 60 °C (VD 60 °C), 80 °C (VD 80 °C); Spray drying (SPD).

The antibacterial activity of PEP against five representative foodborne pathogens (*C. jejuni*, *S. typhimurium*, *E. coli*, *S. aureus*, and *L. monocytogenes*) was evaluated. As shown in Figure 1, all extracts were active against at least one of the pathogens studied, and their antibacterial activity was related to the drying procedure used for their conservation. PEP exhibited different levels of growth inhibition against the foodborne pathogens evidencing a strain-dependent effect. Among all the studied powders, the most relevant antibacterial activity was observed for FD extract. In fact, this extract inhibited significantly (*p* < 0.05) the growth of all the bacteria studied except *E. coli* strain. This extract showed a growth inhibition range between 22–52%, depending on the bacterial strain. VD 60 °C extract inhibited the growth of three of the five foodborne bacterial strains (*C. jejuni*, *S. aureus*, and *L. monocytogenes*), compressing a growth inhibition range of 17–36%. VD 40 °C and VD 80 °C inhibited the growth of two bacterial strains (*C. jejuni*-*L. monocytogenes*, and *E. coli*-*L. monocytogenes*) with an inhibition range of 46–58%, and 26–46%, respectively. Otherwise, SPD extract inhibited only the growth of *L. monocytogenes* strain (17% of inhibition). Taking into account the nature of the microorganism, the results showed that *L. monocytogenes* was inhibited by all the PEP in the range of 17%–46%, regardless of the drying process used (Figure 1).

*C. jejuni*, the leading cause of bacterial foodborne diarrheal illness worldwide, was also inhibited for three of the powders used (FD, VD 40 °C, and VD 60 °C) in a range of 22–58%. FD and VD 60 °C (27–36%) affected the growth of *S. aureus*, while *S. typhimurium* and *E. coli* were the bacterial strains with the lowest sensitivity to the all PEP. The different antibacterial activities by PEP against foodborne bacterial strains may be related to the different composition of phenolic compounds of each sample (Table 2), assuming that it is generally accepted that phenolic compounds, present in plant extracts, play a mandatory role in their antibacterial effects [28]. However, as can be deduced from Table 2, it is not the total concentration of phenolic compounds present in the sample, which determines its antibacterial effect, but rather the presence of certain specific polyphenolic compounds in the extract. In this regard, FD was the most active bactericidal extract (Figure 1), showed a significant higher concentration of quercetin-3-*O*-galactoside (hyperoside). Hyperoside is a flavonol glycoside with variety of biological activities, including anti-inflammatory, antioxidant, and antimicrobial activities [29,30,31]. Its antibacterial effect has been demonstrated both, against gram-negative bacteria such as *P. aeruginosa* [32] and against gram-positive bacteria, such as *S. aureus* [33]. The results obtained in this work suggest that the hyperoside could be involved in the antimicrobial effect observed, since this compound has a significantly higher concentration in the powder obtained by FD, while the rest of the phenolic compounds identified are in concentrations similar or lower than those obtained for the rest of the extracts (Table 2). Apparently, the antibacterial effect of PEP used to be more effective against gram-positive bacteria [14]. This behavior is influenced by differences in the cell membrane constituents. Gram-positive bacteria contain an outer peptidoglycan layer, which is an ineffective permeability barrier; meanwhile gram-negative bacteria have outer phospholipidic membrane carrying structural lipopolysaccharide components, which represent an obstacle for polyphenolic compounds to enter the cell cytoplasm [14,34]. This pattern is also observed in our work, except for *C. jejuni*. Although it is a gram-negative bacterium, *Campylobacter* lacks many of the genetic regulatory networks found in other gram-negative bacteria that allow them to respond to, and cope with, adverse conditions [35]. Accordingly, we have previously demonstrated that *Campylobacter* can be significantly inhibited by different polyphenolic compounds [22,36]. Therefore, antibacterial activity of PEP could be modulated depending of the drying procedure used. The drying process involves several variables, which can change the polyphenolic composition of the extract, resulting in a modified antibacterial response.

### 3.4. Antioxidant Activity Against Intracellular Reactive Oxygen Species (ROS) Production

**Figure 2:**
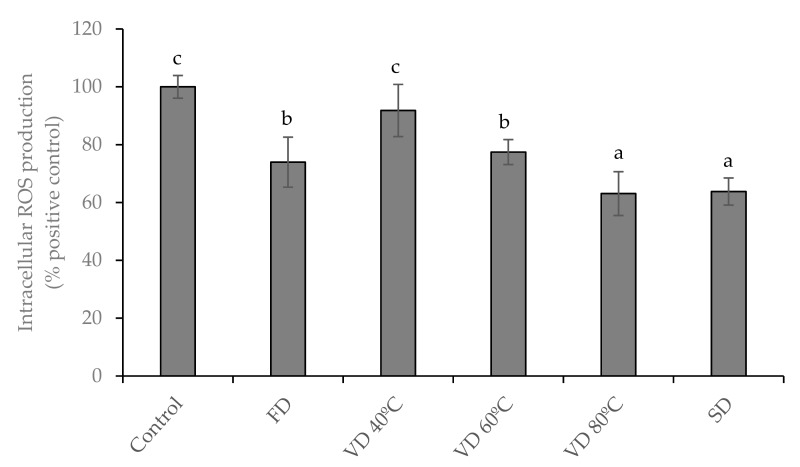
Protective effect of PEP (1 mg/mL) on intracellular ROS production. HT-29 cells were incubated with the powders for 24 h and then treated with 2.5 mM TBHP for 3 h, and ROS production was determined. Values are expressed as a percentage relative to the control conditions and are represented by mean ±SD (*n* = 3). Bars with different letters indicate significant differences on ROS production by ANOVA post hoc LSD Tukey test (*p* ≤ 0.05). Freeze drying (FD); Vacuum drying 40 °C (VD 40 °C), 60 °C (VD 60 °C), 80 °C (VD 80 °C); Spray drying (SPD).

Oxidative stress is involved in several acute and chronic pathological processes due to its ability in activating inflammatory pathways [37,38]. The antioxidant activity of plum polyphenolic compounds has been previously investigated in different system models, such as ABTS, DPPH, FRAP, and ORAC assays [5,16]. However, due to the absence of information on the scavenger activity of ROS in biological models, we investigated the ability of PEP in reducing intracellular oxidative stress. The experiment was carried out using similar concentrations of PEP to those used in the antibacterial activity assay (1 mg/mL). This concentration did not significantly affect cell viability at 24 h after treatment (data not shown). As shown in Figure 2, HT-29 cells, exposed to TBHP, increased the ROS level (oxidative control). Pre-treatment of intestinal cells with PEP showed a significant (*p* < 0.05) reduction in cellular ROS generation, stimulated by TBHP, compared with the oxidative control, except when the cells were pre-treated with VD 40 °C sample. VD 80 °C and SPD caused the highest protection against oxidative damage in stressed cells, inducing an inhibition percentage of ROS production close to 37% respect to the oxidative control cells. Although, FD and VD 60 °C had a significant antioxidant activity with respect to the experimental control, this was lower than the samples obtained with treatments carried out at higher temperatures. Previously, others have shown that drying processes can influence the antioxidant capacity of the dried products, observing an increase in the antioxidant capacity of plum extracts dried by microwave vacuum at high temperatures [3]. Apparently, the temperature applied during plum juice drying is related to the increase in the content of polyphenolic compounds able to scavenge ROS, as the highest values of those compounds were noted after drying at 80 °C. In this regard, the drying temperature of the PEP and the subsequent polyphenolic composition affected the antioxidant capacity of the obtained powders, therefore, the extracts exposed to higher temperatures during the drying process showed a higher content in the methyl 3-caffeoylquinate (Table 2) and significantly improved antioxidant activities. It is well known that phenolic acids can prevent oxidative damage because of their ability to scavenge ROS [39]. Within the phenolic acids, chlorogenic acid and their derivatives, such as methyl 3-caffeoylquinate acid, act as potent ROS scavengers by donating hydrogen atoms to reactive molecules, transforming them to less active radicals, and maintaining an optimal cellular oxidative balance [40,41,42].

Our findings show that PEP could induced cellular protection against TBHP-induced oxidative stress by preventing intracellular ROS accumulation. However, the level of antioxidant capacity of the PEP may be determined by the conditions applied during the drying process.

### 3.5. Anti-Inflammatory Activity

**Figure 3:**
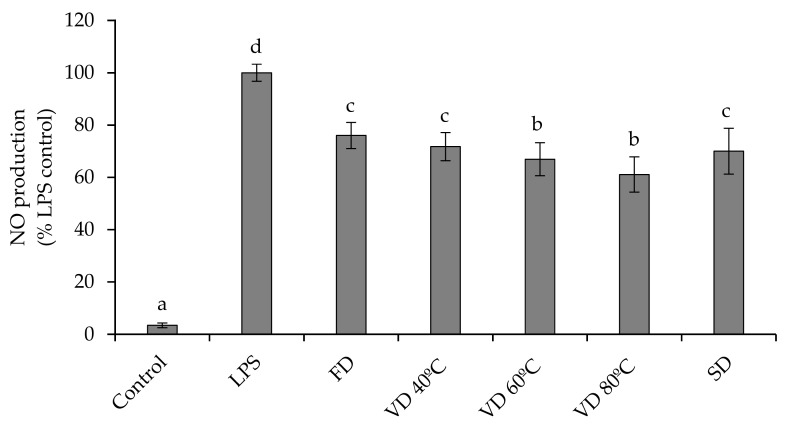
Effect of PEP (1 mg/mL) on nitric oxide (NO) production in LPS-stimulated RAW264.7 macrophage cells. Values are expressed as a percentage relative to the LPS-stimulated control group and are represented by mean ±SD (*n* = 3). Bars with different letters indicate significant differences on NO production by ANOVA LSD Tukey test (*p* ≤ 0.05). Freeze drying (FD); Vacuum drying 40 °C (VD 40 °C), 60 °C (VD 60 °C), 80 °C (VD 80 °C); Spray drying (SPD).

We investigated the impact of PEP on the inflammatory process in RAW264.7 cells stimulated with LPS. During an inflammatory event, the inducible enzyme nitric oxide synthase (iNOS) is responsible for an exacerbated production of NO, which can lead to tissue lesions, organ dysfunction, and inflammation-related diseases [43]. As shown in Figure 3, LPS significantly (*p* < 0.05) stimulated the production of NO in macrophages (Control+) with respect to non-stimulated cells (Control−). The percentage of NO production was significantly (*p* < 0.05) reduced by 24–39% in RAW264.7 cells pre-treated with PEP. Pre-treatment of LPS-stimulated cells with VD 80 °C and VD 60 °C powders led to greater attenuation of NO production with an inhibition range between 33% and 39%. The rest of the extracts had a similar behavior (*p* < 0.05), regardless of the drying treatment conducted, and caused an attenuation in the production of NO in a range between 24% and 30%. Apparently, a greater anti-inflammatory capacity would be influenced both, by the type of treatment used (VD > SPD > FD) and by the temperature used in each treatment. Overall, this behavior is similar to that observed in the results obtained from the analysis of antioxidant activity (ROS inhibition), where VD 80 °C was the most active sample in both determinations. Some investigators have demonstrated that the cellular inflammatory responses are caused because of ROS production [17,44]. Consequently, a reduction in intracellular ROS levels could lead to an inhibition of the inflammation process through the reduction of NO production. This postulate agrees with our results, since the samples that showed high antioxidant activity by reducing intracellular ROS (Figure 2) also showed relevant anti-inflammatory properties by reducing NO production (Figure 3).

However, it is difficult to precise the main polyphenolic compounds that would be most involved in the anti-inflammatory response observed, although apparently, several of them appear to be involved. Previous studies have demonstrated that the anti-inflammatory effects of plum phenolic compounds [1,18], mainly due to the presence of phenolic acids, can decrease the expression of inflammatory mediators, such as nuclear factor κB (NF-κB), vascular cell adhesion molecule 1 (VCAM-1), cyclooxygenase-2 (COX-2), and iNOS mRNA [45]. In addition, plum phenolic compounds can contribute to the modulation of the inflammatory responses in human cells by inhibiting various inflammatory factors, such as cytokines IL-6 and IL-8 [17]. In addition, the potential anti-inflammatory activity of the PEP could be particularly important, considering that activation inflammatory pathways can stimulate proliferation of cancer cells [38].

## 4. Conclusions

This study has shown that plum extract powders gained after freeze-, vacuum-, and spray-drying have promising antibacterial, antioxidant, and anti-inflammatory properties that have been tested in different biological models. The drying processes significantly influences both, the physical properties and the composition of polyphenols, and thus, the bioactive properties plum juice extract powders. The drying techniques moderated the content of polyphenolic compounds in the powders, which influence the different levels of growth inhibition against the foodborne pathogens, evidencing a strain-dependent effect being the most relevant for FD extract. This extract inhibited significantly (*p* < 0.05) the growth of all the bacteria studied, except *E. coli* strain. It was observed that *L. monocytogenes* was inhibited by all the dried plum juice extracts in the range of 17–46%, regardless of the drying process used. These powders could also induce cellular protection against oxidative stress by preventing intracellular ROS accumulation, but the level of antioxidant capacity may be determined by the conditions applied during the drying process. It was shown that VD 80 °C and SPD caused the highest protection against oxidative damage in stressed cells, inducing an inhibition percentage of ROS production close to 37% respect to the oxidative control cells. Moreover, plum extracts powders exhibited a greater anti-inflammatory capacity, which would be influenced both, by the type of treatment used and by the temperature used in each treatment, being the VD 80 °C - the sample with the highest protection level. The results demonstrate that the drying method selected can be an effective tool for modulating the composition, physical, and bioactive properties of plum extracts powders.

## Figures and Tables

**Table 1:** Physical properties of the plum extracts powders (PEP) obtained after different drying techniques.

	Moisture Content (%)	Water Activity (-)	Colour				
			L*	a*	b*	C*	h*
FD	7.34 ± 0.14 ^d^	0.289 ± 0.001 ^d^	42.84 ± 0.13 ^b^	23.37 ± 0.1 ^d^	10.73 ± 0.03 ^d^	25.72 ± 0.10 ^c^	24.67 ± 0.13 ^b^
VD 40 °C	3.74 ± 0.17 ^c^	0.227 ± 0.002 ^b^	40.28 ± 0.05 ^a^	17.73 ± 0.04 ^c^	7.64 ± 0.01 ^b^	19.31 ± 0.03 ^b^	23.33 ± 0.05 ^a^
VD 60 °C	7.17 ± 0.04 ^d^	0.243 ± 0.001 ^c^	43.10 ± 0.67 ^b^	16.94 ± 0.61 ^b^	8.33 ± 0.10 ^c^	18.88 ± 0.59 ^b^	26.19 ± 0.56 ^c^
VD 80 °C	3.29 ± 0.08 ^b^	0.242 ± 0.001 ^c^	40.40 ± 0.09 ^a^	13.56 ± 0.02 ^a^	6.64 ± 0.02 ^a^	15.11 ± 0.03 ^a^	26.09 ± 0.07 ^c^
SPD	2.44 ± 0.06 ^a^	0.146 ± 0.002 ^a^	45.76 ± 0.02 ^c^	24.97 ± 0.06 ^e^	13.15 ± 0.03 ^e^	28.22 ± 0.04 ^d^	27.78 ± 0.09 ^d^

Freeze drying (FD); Vacuum drying 40 °C (VD 40 °C), 60 °C (VD 60 °C), 80 °C (VD 80 °C); Spray drying (SPD); colour parameters (L*, a*, b*), chroma (C*) and hue (*h*). Different letters (a,b,c,d,e) within the same column indicated statistical differences between samples (*p* ≤ 0.05; LSD Tukey).

**Table 2:** Content of identified major phenolic compounds in plum extracts powders (PEP) obtained by selected drying methods (g/100 g db).

Compound	FD	VD 40 °C	VD 60 °C	VD 80 °C	SPD
**Phenolic acids**					
Neochlorogenic acid	10.25 ± 0.12 ^c^	10.18 ± 0.02 ^c^	8.85 ± 0.11 ^b^	6.45 ± 0.02 ^a^	11.04 ± 0.33 ^c^
3-feruoylquinic acid	10.51 ± 0.06 ^c^	9.96 ± 0.11 ^c^	8.51 ± 0.36 ^b^	5.42 ± 0.15 ^a^	11.69 ± 0.28 ^d^
3-*O*-*p*-coumaroylquinic acid	4.41 ± 0.09 ^ab^	4.73 ± 0.11 ^b^	4.59 ± 0.15 ^ab^	4.05 ± 0.21 ^a^	5.30 ± 0.14 ^c^
Chlorogenic acid	4.14 ± 0.01 ^c^	3.96 ± 0.01 ^bc^	3.58 ± 0.02 ^ab^	3.36 ± 0.12 ^a^	4.70 ± 0.04 ^d^
Methyl 3-caffeoylquinate	0.25 ± 0.01 ^a^	0.37 ± 0.01 ^ab^	0.55 ± 0.02 ^b^	7.08 ± 0.11 ^d^	3.92 ± 0.06 ^c^
Total phenolic acids	29.56	29.20	26.08	26.36	36.65
**Flavonols**					
Quercetin-3-*O*-rutinoside	1.25 ± 0.01 ^c^	0.09 ± 0.01 ^a^	0.24 ± 0.04 ^b^	1.09 ± 0.16 ^c^	1.88 ± 0.17 ^d^
Quercetin-3-*O*-galactoside	5.01 ± 0.05 ^d^	1.29 ± 0.01 ^b^	1.41 ± 0.01 ^c^	1.47 ± 0.06 ^c^	0.73 ± 0.04 ^a^
Quercetin-3-*O*-glucoside	0.73 ± 0.01 ^a^	5.24 ± 0.02 ^c^	5.14 ± 0.07 ^c^	4.58 ± 0.09 ^b^	6.11 ± 0.14 ^d^
Quercetin-3-*O*-(6′′acetylgalactoside)	1.03 ± 0.01 ^c^	1.04 ± 0.01 ^c^	0.89 ± 0.11 ^b^	0.61 ± 0.01 ^a^	1.21 ± 0.09 ^d^
Total flavonols	8.02	7.66	7.68	7.75	9.93
**Anthocyanins**					
Cyanidin-3-*O*-glucoside	0.27 ± 0.02 ^c^	0.26 ± 0.01 ^c^	0.24 ± 0.04 ^b^	0.17 ± 0.03 ^a^	0.28 ± 0.01 ^c^
Cyanidin-3-*O*-rutinoside	0.70 ± 0.01 ^bc^	0.70 ± 0.00 ^bc^	0.66 ± 0.11 ^ab^	0.53 ± 0.03 ^a^	0.78 ± 0.04 ^bc^
Peonidin-3-*O*-rutinoside	0.0058 ± 0.0001 ^b^	0.0058 ± 0.0002 ^b^	0.0055 ± 0.0009 ^b^	0.0044 ± 0.0007 ^a^	0.0065 ± 0.0003 ^c^
Total anthocyanins	0.97	0.96	0.90	0.70	1.07
**Total polyphenolics content**	38.55	37.82	34.66	34.81	47.65

Freeze drying (FD); Vacuum drying 40 °C (VD 40 °C), 60 °C (VD 60 °C), 80 °C (VD 80 °C); Spray drying (SPD); different letters (a,b,c,d) within the rows indicate significant differences between samples (*p* ≤ 0.05; LSD Tukey test).
